# GABAergic striatal neurons project dendrites and axons into the postnatal subventricular zone leading to calcium activity

**DOI:** 10.3389/fncel.2014.00010

**Published:** 2014-01-28

**Authors:** Stephanie Z. Young, Carlos A. Lafourcade, Jean-Claude Platel, Tiffany V. Lin, Angélique Bordey

**Affiliations:** ^1^Departments of Neurosurgery and Cellular and Molecular Physiology, Yale University School of MedicineNew Haven, CT, USA; ^2^Developmental Biology, Aix-Marseille University, IBDML, CNRS, UMR7288Marseille, France

**Keywords:** GABA, striatum, calcium activity, neurogenesis, proliferation, medium spiny neurons, Parkinson disease, huntington disease

## Abstract

GABA regulates the behavior of neuroblasts and neural progenitor cells in the postnatal neurogenic subventricular zone (SVZ) through GABA_A_ receptor (GABA_A_R)-mediated calcium increases. However, the source of GABA necessary for sufficient GABA_A_R-mediated depolarization and calcium increase has remained speculative. Here, we explored whether GABAergic striatal neurons functionally connect with SVZ cells. Using patch clamp recordings or single cell electroporation, striatal neurons along the SVZ were filled with a fluorescent dye revealing that they send both dendrites and axons into the SVZ. About 93% of the recorded neurons were medium spiny or aspiny GABAergic neurons and each neuron sent 3–4 processes into the SVZ covering ~56 μm. Using calcium imaging, we found that depolarization of striatal neurons led to increased calcium activity in SVZ cells that were mediated by GABA_A_R activation. Collectively, these findings undercover a novel mode of signaling in the SVZ providing a mechanism of brain activity-mediated regulation of postnatal neurogenesis through GABAergic striatal activity.

## Introduction

Neural progenitor cells (NPCs) persist in the adult brain throughout life in all animal species examined, including humans (Bonfanti and Peretto, 2011). The largest region is the subventricular zone (SVZ) along the lateral ventricle that spans the entire cerebrum. In the SVZ, NPCs and neurogenesis are influenced by a large repertoire of intracellular and extracellular molecules (Pathania et al., 2010). One of the molecules that regulates every step of neurogenesis is the amino acid γ-aminobutyric acid (GABA), which is the main inhibitory neurotransmitter in the adult brain.

In the SVZ, GABA acts as a local neurotransmitter and regulates NPC proliferation as well as neuroblast proliferation and migration through GABA_A_ receptors (GABA_A_Rs) (Nguyen et al., 2003; Bolteus and Bordey, 2004; Liu et al., 2005; Mori et al., 2006; Cesetti et al., 2010; Young et al., 2012). GABA is thought to act as a paracrine neurotransmitter that is released from neuroblasts in a non-vesicular mechanism and provides tonic GABA_A_R activation (Bolteus and Bordey, 2004; Liu et al., 2005). However, such tonic receptor activation does not fit with recent data showing that GABA_A_R-regulated NPC proliferation requires a large phasic release of GABA to depolarize NPCs above the threshold for opening voltage-gated calcium channels leading to calcium influx (Bordey, 2007; Young et al., 2012). The necessity for such a phasic and perhaps synaptic source of GABA onto SVZ cells had been previously hypothesized (Bordey, 2006), but has never been examined. Intriguingly, the SVZ is located adjacent to the striatum and nucleus accumbens. More than 90% of the striatal neurons are GABAergic (Ostergaard, 1993; Kawaguchi et al., 1997; Tepper et al., 2004) and are thus in perfect location to alter cell development in the SVZ. However, whether striatal inputs project into the SVZ remains unknown.

Here, to address this issue, we filled striatal neurons located along the postnatal SVZ with fluorescent dyes in acute brain slices. We found that striatal GABAergic neurons send both dendrites and axons into the SVZ reaching out to neuroblasts and glial fibrillary acidic protein (GFAP)-positive cells, some of which are NPCs (Doetsch et al., 1999). In addition, neuronal depolarization leads to GABA_A_R-mediated calcium activity in SVZ cells. These findings provide a mechanism for activity-dependent induction of GABA_A_R-mediated calcium activity in SVZ cells that is known to regulate NPC proliferation (Young et al., 2012) associated with local blood flow increase (Lacar et al., 2012a,b). Striatal activity may thus coordinate SVZ cell proliferation and blood-related metabolic supplies providing a mechanism of brain activity coupling to neurogenesis (Parent et al., 2002, 2006; Young et al., 2011). These data add to the already known mechanism of neuronal nitric oxide and dopamine regulations of SVZ neurogenesis (Moreno-Lopez et al., 2000, 2004; Baker et al., 2004; Freundlieb et al., 2006; Winner et al., 2006; O'Keeffe et al., 2009) [for review (Young et al., 2011)].

## Materials and methods

### Animals

Experiments were performed in wild type CD1 mice (Charles River) and several lines of transgenic mice: (1) homozygote mice carrying GFP under the doublecortin promoter (*Dcx*-GFP mice, FVB/N strain, a gift from Dr. R. Miller, University of Chicago, originally from Gensat) and (2) human glial fibrillary acidic protein promoter encoding GFP (*hGfap*-GFP mice, Jackson Labs), and (3) transgenic *hGfap-tTA/TetO*-MrgA1:GFP mice (*hGfap*-MrgA1:GFP mice, a gift from Dr. Ken McCarthy, University of North Carolina at Chapel Hill). In the absence of doxycycline, GFAP-expressing cells including those in the SVZ express MrgA1 receptors fused to GFP (Fiacco et al., 2007; Platel et al., 2010; Lacar et al., 2012b). All experimental protocols were approved by the Institutional Animal Care and Use Committees of Yale School of Medicine. Mice of either gender were used between postnatal day (P) 14 and P25.

### Acute slice preparation, patch clamp recordings, and single neuron electroporation

Acute coronal or sagittal brain slices (250–300 μm-thick) containing the SVZ were prepared as we previously described (Wang et al., 2003; Bolteus and Bordey, 2004; Platel et al., 2010). Slices were placed in a flow-through chamber and continuously superfused with oxygenated artificial cerebrospinal fluid (aCSF) containing (in mM): NaCl 124; KCl 3; CaCl_2_ 2.5; MgSO_4_ 1.2; NaH_2_PO_4_ 1.23; NaHCO_3_ 26; glucose10. Experiments were performed on an upright Olympus BX61WI microscope equipped with an Olympus FluoView 300 confocal microscope and a water-immersion Nomarski phase-contrast and fluorescence 60X objective (N.A. 0.9).

For whole-cell patch clamp recordings, borosilicate pipettes (Sutter) were pulled on a P-97 sutter puller and had resistances of 4–6 MΩ when filled with an intracellular solution containingthe following: 110 mM KCl, 1.0 mM CaCl_2_, 10 mM EGTA, 10 mM HEPES, 50 μ M Alexa Fluor 488 or 568 dye and an ATP-regenerating solution thatincluded 4 mM K_2_ATP, 20 mM K_2_-phosphocreatine, 50 U/ml creatinephosphokinase, and 6 mM MgCl_2_. The pH and the osmolarity were adjusted to 7.2and 290 mOsm, respectively. The liquid junctionpotential (~4 mV) was not corrected. Whole-cell recordings were performed using an Axopatch 200B amplifier, and current signals were low-passfiltered at 2–5 kHz and digitized on-line at 5–20 kHz usinga Digidata 1320 digitizing board (Axon Instruments, Foster City,CA). Recorded neurons were held at −70 mV close to their resting potential of −70 to −75 mV. Capacitive and leak currents were not subtracted.

Single cell electroporation was performed with a thin glass electrode (OD: 1.5; ID: 1.10, 8–15 MΩ) pulled on a Sutter P-97 pipette puller and filled with either intracellular recording solution or 125 mM KCL solution. We used an Axoporator 800A to apply square pulse of −7 to −8 V at 100–150 Hz for 1 s duration (0.0V offset).

### Immunohistochemistry

Slice preparation, immunostaining, and image acquisition and analysis were as previously described (Platel et al., 2009). Primary antibodies included: anti-VGAT1 (mouse, 1:500, Synaptic Systems), anti-neurofilament (NF, 1:500, MAB5166) anti-DCX (goat or rabbit, 1:100, Santa Cruz, SC8066 and SC28939), and anti-GLAST (guinea pig, 1:500, Chemicon). Each staining was replicated at least in 4–5 slices from three different mice. The appropriate secondary antibodies were Alexa fluor series (1:1000, Invitrogen, USA) or Cyanine series (1:500, Jackson Labs). Z-section images (spaced by 1–2 μm over 10–20 μm) were acquired on a laser-scanning confocal microscope (Olympus FluoView 1000) with a 20× dry objective (N.A. 0.75) or a 60× oil objective (N.A. 1.42). Images were analyzed and reconstructed using Imaris 4.0 (Bitplane AG, Switzerland) and Photoshop CS3 (Adobe, USA).

### Neuron reconstruction

Neuronal processes in confocal *Z*-stack were traced using Simple Neurite Tracer in FIJI (NIH ImageJ 1.39t) (Schindelin et al., 2012).

### Dye loading of SVZ cells and calcium imaging

SVZ cells were loaded by pressure application of Fluo-4 AM (100 μ M in aCSF, 0.4% Pluronic acid F-127, Invitrogen). Occasionally SVZ cells were loaded with CellTracker Orange (Molecular Probes) pressured applied at 100 μM. Images were acquired every 1 s with FluoView acquisition software. For spontaneous movies, images were acquired every 2 s (0.5 Hz). F_0_ (i.e., baseline) and F are the mean fluorescence intensities measured throughout all of the regions of interest (ROIs) and in each ROI, respectively. A change in fluorescence was considered to be a Ca^2+^ increase if it was >15% F/F_0_ increase. Intracellular Ca^2+^ changes were calculated using Calsignal (Platel et al., 2007) and Clampfit 10. F/F_0_ was detected with Calsignal and traces were exported into Clampfit for peak analysis using the threshold detection function. For peak analysis, the baseline for each ROI trace was manually adjusted to zero. In addition, traces from control and drug-treated movies were concatenated and the same threshold for peak detection was used_._ Bicuculline methiodide and tetrodotoxin were from Tocris Biosciences (MO, USA).

### Statistics

Data are expressed as mean ± standard error. Statistical analysis used a two-tailed Student's *t*-test or One-Way ANOVA.

## Results

### Vesicular GABA transporters are expressed in the SVZ

**Figure 1:**
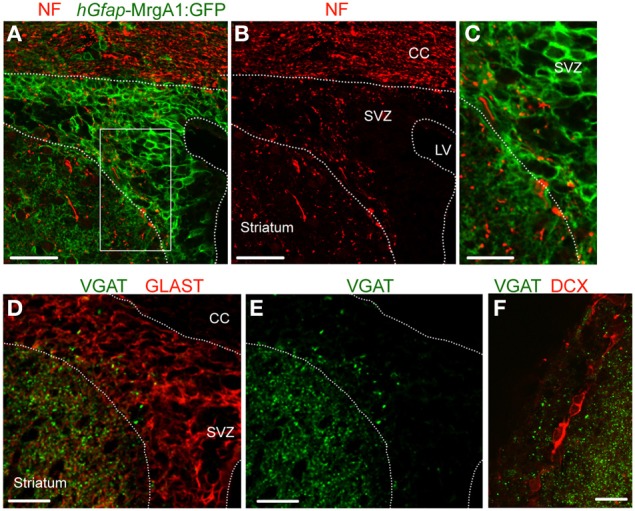
**VGAT is expressed in the SVZ close to GFAP-positive cells and neuroblasts. (A,B)** Confocal images of neurofilament immunostaining (NF, red, A) and GFP expression (green, **A,B**) in the SVZ in a coronal section from a *hGfap*-MrgA1:GFP mice. **(C)** Zoom from the white square shown in **(A)**. **(D,E)** Confocal images of VGAT immunostaining (green, **D,E**) and GLAST (red, **D**), which decorates GFAP-expressing cells. GLAST is a glutamate transporter. **(F)** Confocal images of VGAT immunostaining (red) and DCX-expressing cells, neuroblasts (red). Scale bars: 40 μm **(A,B)**, 20 μm **(C,F)**, and 25 μm **(D,E)**. CC, corpus callosum; LV, lateral ventricle.

Previous studies reported the presence of axonal terminals in the SVZ (Peretto et al., 1999; Moreno-Lopez et al., 2000; Mercier et al., 2002; Freundlieb et al., 2006), we thus immunostained for neurofilament (NF), an axonal marker. Punctate NF immunostaining (red) was indeed found in the SVZ that was apposed to GFP-positive processes (green) belonging to GFAP-expressing cells (Figures 1A–C). To examine for GABA release sites, we immunostained for vesicular GABA transporter (VGAT), which is expressed in GABA-containing vesicles of GABAergic synaptic terminals. VGAT immunostaining (green) was observed in the SVZ (Figures 1E,F), and was apposed to GLAST-positive processes of GFAP-positive cells (Figure 1D) and doublecortin (DCX)-positive neuroblasts (Figure 1F), but was excluded from these cell types. These data suggest the presence of GABAergic terminals in the SVZ.

### Striatal neurons project dendrites and axons into the SVZ

**Figure 2:**
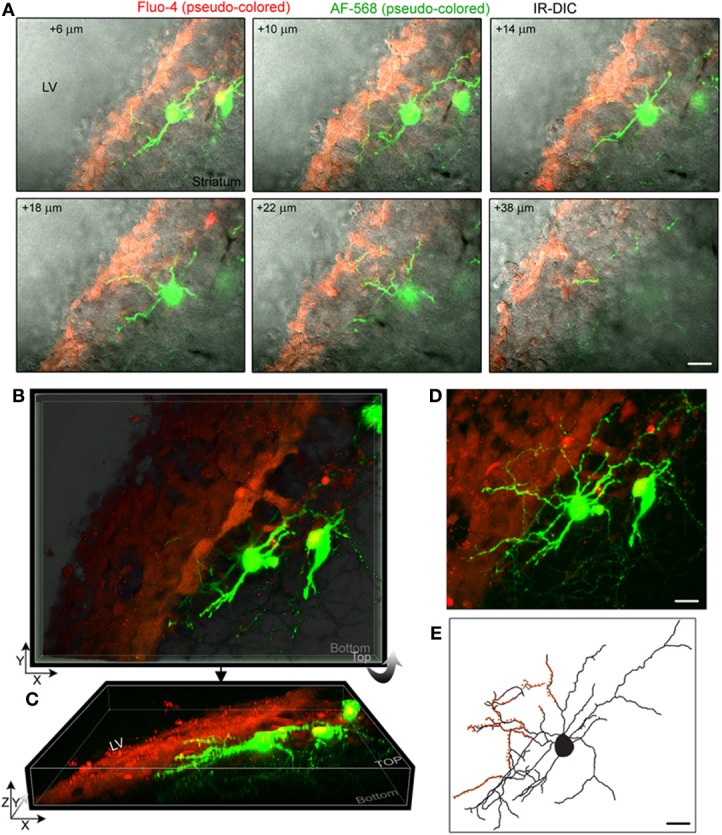
**Striatal neurons project dendrites into the SVZ. (A)** Images of single optical sections at different depths (indicated in the right corner) from the surface of the sagittal slice. Two striatal neurons were filled with Alexa Fluor-568 (pseudo-colored green) and the fluorescent dye Fluo-4AM (pseudo-colored red) was pressure applied in the SVZ. Not every SVZ cell is Fluo-4-positive. Images are overlaid on the IR-DIC. **(B)** 3D Z-projection using the blend mode in Imaris illustrating the 3D shape of the SVZ in a sagittal section. **(C)** 3D Y-projection of the image in B following a rotation up around the X axis. **(D)** Maximum intensity projection in Imaris. **(E)** Rreconstruction of one of the filled neuron with orange overlaid to marked the processes entering the SVZ. Scale: 30 μm **(A,D,E)**.

**Figure 3:**
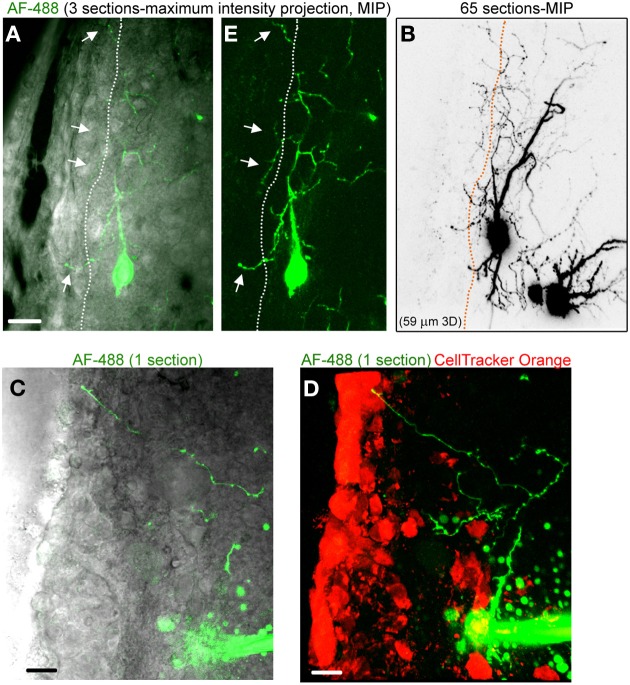
**Striatal neurons project axons into the SVZ. (A)** Images of a three section-MIP of a AF-488-filled neuron with and without IR-DIC overlay. The arrow points to processes entering the SVZ. **(B)** Image of a 65 section-MIP illustrating the processes entering the SVZ. The processes in the SVZ resemble axons, which display varicosities and are much thinner than dendrites. **(C,D)** A one section-image of a AF-488-labeled neuron overlaid with IR-DIC and a five section-MIP image of the neuron overlaid with CellTracker Orange. The neuron sends an axon into the SVZ. Scales: 20 μm **(A)** and 10 μm **(C,D)**.

To examine whether striatal neurons send processes into the SVZ, we used single cell electroporation or patch clamp recording to label neurons with the fluorescent dye Alexa Fluor 488 (green) or Alexa Fluor 568 (red) in acute sagittal or coronal sections. Recorded neurons were at a maximum of 40 μm away from the SVZ (Figure S1). The SVZ was identified with either loading with the calcium indicator dye Fluo-4 AM (green) applied by pressure (Figure 2A), with the vital dye CellTracker orange (Figure S1), or simply by examining the infrared differential interference contrast (IR-DIC) image (Figures 2, 3).

**Figure 4:**
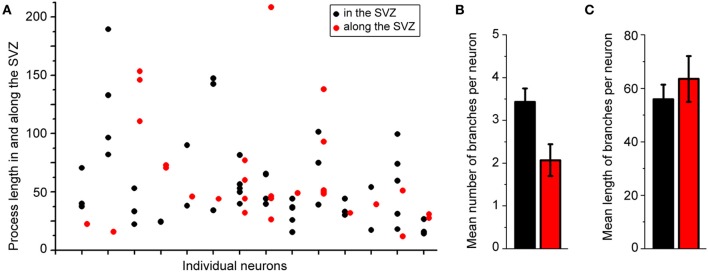
**About 80% of the labeled striatal neurons send one or two processes into the SVZ. (A)** Plots illustrating the length of processes in the SVZ from individual neurons. **(B,C)** Mean number **(B)** and mean length **(C)** of neuronal processes in the SVZ.

Figure 2A displays several Z-sections of an AF-488-labeled neuron (pseudo-colored green) located along the SVZ in a sagittal slice. Processes projecting into the SVZ are visible in the different sections. The calcium indicator dye Fluo-4 applied by pressure does not equally label every SVZ cell due to the cell density in this region, resulting in gap in labeling (Lacar et al., 2010, 2012b). Figure 2B represents a Z- and a Y-projection in a blending perspective mode illustrating the lateral wall and two neurons recorded in the striatum and allowing to visualize the 3D structure of the SVZ in a sagittal slice. A maximum intensity projection and a reconstruction of the neuron illustrate the extent of processes entering the SVZ (Figures 2D,E). Surprisingly, the majority of the processes entering the SVZ resembled dendrites, which are thicker than axons and without varicosities (Figure 2D). Figure S1 displays another AF-488-labeled neuron sending dendrites into the SVZ. Processes resembling axons that reached and entered the SVZ could also be seen from filled neurons (Figure 3). Due to their thickness, dendrites were easier to visualize than axons leading to a presumed bias toward visualization of dendrites into the SVZ. As a result, for the purpose of quantification, we did not distinguish between axons or dendrites. Out of 132 labeled neurons, we found that 78% of the filled neurons send processes into the SVZ. The average number of processes per neuron entering the SVZ was 3.5 and covered a mean distance of 56 μm in the SVZ and 63 μm along the SVZ (*n* = 14 neurons analyzed, black and red, respectively on graphs, Figure 4).

Collectively, these data show the presence of processes, including dendrites and axons from striatal neurons projecting into the SVZ.

### Processes of striatal neurons are apposed to GFAP-positive cells and neuroblasts

**Figure 5:**
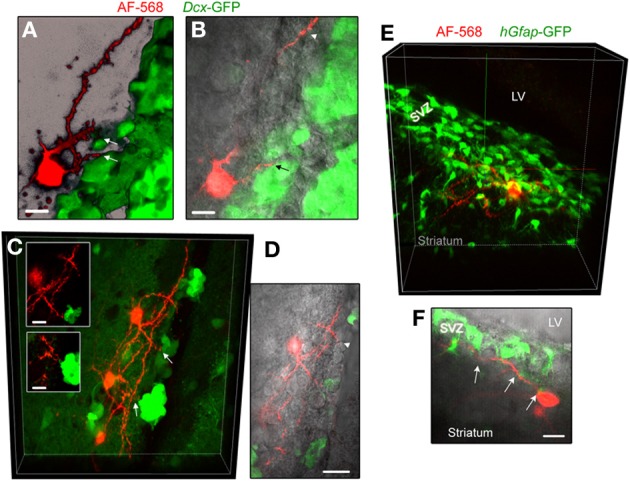
**Striatal neurons send processes toward neuroblasts and GFAP-expressing cells. (A,B)** Images of an AF-568-filled striatal neuron sending a process toward neuroblasts that are GFP-positive in *Dcx*-GFP mice. A represents a 3D projection in the blend mode of 21 sections (Z of 17 μm) and B represents 10 sections overlaid with IR-DIC. **(C)** Maximum intensity projection of three AF-568-labeled neurons in a coronal section from a *Dcx*-GFP mouse. Insets: zoom of the processes projecting toward neuroblasts (arrows in the main image) in one optical section. **(D)** Image of one optical section of the AF-568-labeled neuron and GFP fluorescence shown in **(C)** overlaid with IR-DIC image. The IR-DIC illustrates that neuronal processes also project toward GFP-negative cells. **(E)** Maximum intensity projection of a AF-568-labeled neurons in a sagittal section from a *hGfap*-GFP mice. The recorded striatal neuron sits below the SVZ and send a few processes into the SVZ. **(F)** Image of one optical section of the AF-568-labeled neuron and *hGfap*-GFP fluorescence shown in **(E)** overlaid with IR-DIC image. Arrows point to sites of contact between the neuronal processes and GFP-positive cell processes. Scale bars: 10 μm **(A,B,C)** and 15 μm **(F)**.

In an effort to examine whether neuronal processes contact neuroblasts or GFAP cells, the two major cell types in number in the SVZ, we filled neurons in transgenic mice expressing GFP under the *Dcx* promoter or the human *Gfap* promoter. In slices from *Dcx*-GFP mice, we found that AF-568-filled neurons sent processes toward GFP-positive cells, neuroblasts (Figures 5A–D). Although these processes were in close apposition with neuroblasts, they did not appear to directly touch neuroblasts (Inset in Figure 5C). In addition, processes were projecting toward *Dcx*-GFP-negative cells. In slices from *hGfap*-GFP mice, fluorescently labeled neurons send processes in close apposition with processes from GFAP-positive cells (Figures 5E,F). Ultrastructural analysis would be required to further examine the connection between neuronal processes and GFAP-positive cells in the SVZ.

### Neurons projecting into the SVZ are GABAergic

**Figure 6:**
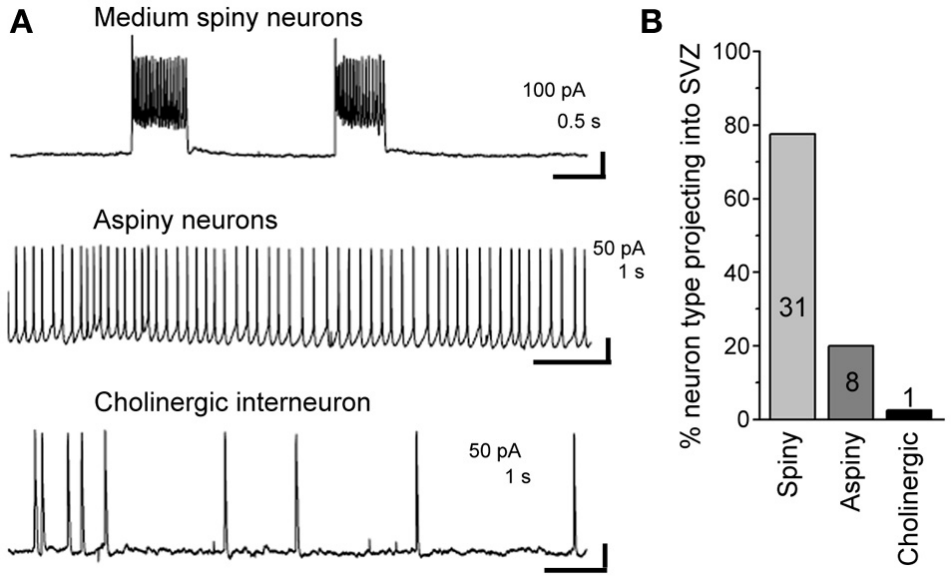
**About 98% of striatal neurons projecting into the SVZ are GABAergic. (A)** Representative recordings of the three different neuronal types sending processes into the SVZ. **(B)** Bar graph of the % of neuronal type contacting the SVZ. The number in each bar represents the number of recorded neurons.

Using patch clamp recordings, we identified three types of neurons contacting the SVZ. This included medium spiny neurons displaying bursts of firing, aspiny neurons displaying pacemaker activity, and cholinergic neurons with irregular firing activity (Figure 6A). Spiny and aspiny neurons are known to be GABAergic (Ostergaard, 1993; Kawaguchi, 1997; Tepper et al., 2004). Aspiny neurons and cholinergic neurons could also be differentiated based on their soma size with cholinergic neurons displaying a much larger soma size than aspiny neurons. Based on the electrophysiology, we found that the majority of neurons projecting to the SVZ were GABAergic spiny (77.5%) and aspiny (20%) (*n* = 40 neurons, Figure 6B).

### Firing of striatal neurons increases calcium activity in SVZ cells

**Figure 7:**
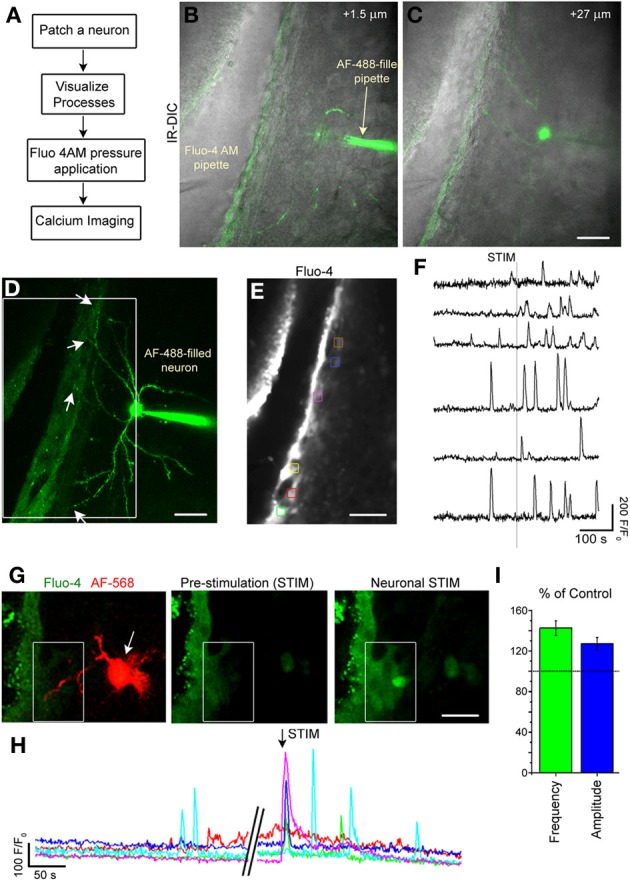
**Single neuron stimulation increases calcium activity in SVZ cells. (A)** Diagram of experimental protocol. **(B,C)** Image of an optical section of a AF-488-filled neuron sending projection into the SVZ. **(D)** Maximum intensity projection of the AF-488-filled neurons whose single sections are shown in **(B,C)**. **(E)** Fluo-4 AM loaded SVZ cells. The colored boxes illustrate regions of interest (ROIs) analyzed for calcium activity. **(F)** Calcium activity graphs from ROIs shown in **(E)**. **(G)** Fluo-4 loaded SVZ cells and AF-488-filled neuron (1 optical section) prior to neuronal stimulation and during neuronal stimulation. **(H)** Calcium activity graphs for the cells in the white box drawn in **(G)**. **(I)** Bar graphs of the percent (%) of control for the frequency (green) and amplitude (blue) of calcium transients during and 2 min following neuronal stimulation compared to the pre-stimulation period. Scale bars: 30 μm **(B–E)** and 15 μm **(G)**.

To assess whether the neuronal processes in the SVZ could alter SVZ cell activity, we performed calcium imaging (Figure 7A) while depolarizing striatal neurons. Calcium activity, such as calcium transients and calcium waves, is one of the signaling mechanisms for SVZ cells to regulate their behavior in a paracrine and autocrine fashion through the release of diffusible signals such as glutamate, and ATP (Platel et al., 2010; Lacar et al., 2012b). In addition, GABA has been shown to lead to calcium increase in both neuroblasts and GFAP-expressing cells in the SVZ through GABA_A_ receptor activation (Nguyen et al., 2003; Young et al., 2010, 2012).

Striatal neurons were recorded and filled with the AF-488 or −568 fluorescent dye to visualize neuronal processes in the SVZ (Figures 7C,D,G). Then, SVZ cells were loaded with the calcium indicator Fluo-4 AM applied by pressure (Figures 7B,E). Recorded neurons were depolarized to induce a 10–15 Hz burst of firing to mimic *in vivo* firing (Murer et al., 2002). Calcium imaging was acquired at 1–2 Hz in a region where neuronal processes were present. Single-neuron depolarizations in current clamp mode induced Ca^2+^ increases in 80% of the SVZ cells that were located at <60 μm away from the neuronal processes (*n* = 7 neurons, 5 mice, Figures 7F,H) accompanied with an increase in the frequency and amplitude of calcium transients (*n* = 5 neurons, 81 SVZ cells analyzed, Figure 7I).

**Figure 8:**
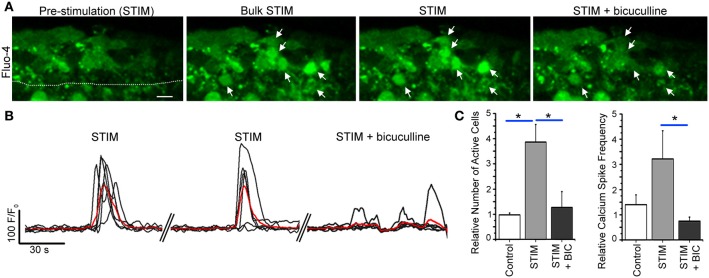
**Bulk striatal stimulation close to the SVZ induces calcium transients in SVZ cells. (A)** Fluo-4 AM loaded SVZ cells prior to and during stimulation without and with bicuculline. Arrows point to cells that responded with a calcium activity during neuronal stimulation that was abolished by bicuculline. The stimulating pipette (not shown) was about 60–70 um away from the SVZ outlined by a dotted line. **(B)** Calcium activity graphs for cells in **(A)**. **(C)** Bar graphs of the relative number of active cells and the frequency of calcium transients prior to and during stimulation without and with bicuculline. Scale bar: 10 μm. ^*^p < 0.05 with One-Way ANOVA.

Because in only ~25% of the cases, the calcium response was able to be elicited repeatedly following single neuron depolarization, we tested the efficiency of bulk stimulation by a bipolar stimulating pipette made of a small diameter borosilicate theta glass pipette filled with bath solution located 40–60 μm from the SVZ. A 20 Hz stimulation for 500 ms elicited repeated calcium responses in SVZ cells associated with an increase in the number of active cells and the frequency of calcium transients per cell (Figures 8A,B). These calcium responses were significantly reduced by bath application of tetrodotoxin (TTX, 1 μM, data not shown) at 1 μM to block action potentials and by the GABA_A_ receptor inhibitor bicuculline (50 μM, *n* = 3 slices, Figure 8C), suggesting an action potential-dependent release of GABA from striatal neurons into the SVZ. Consistent with a low baseline firing rate of GABAergic striatal neuron in slices (Plenz and Kitai, 1998), TTX has no detectable effect on spontaneous calcium transients in SVZ cells (*n* = 3, 4.8 ± 1.5 transients/min control and 5.2 ± 2.1 transients/min, *p* = 0.87, data not shown). Nevertheless, we previously reported a significant effect of bicuculline on baseline calcium transients (Young et al., 2010). These findings are addressed in the discussion in light of the dual source of GABA onto SVZ cells.

## Discussion

**Figure 9:**
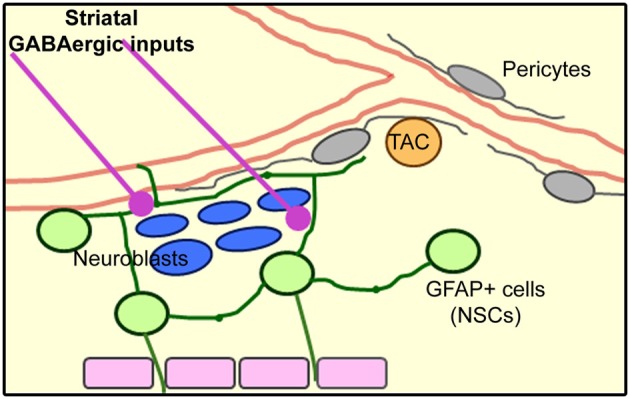
**Model of the SVZ cellular architecture based on published results and the novel identification of striatal inputs into the SVZ**.

Here, we identified the presence of dendrites and axons from mainly GABAergic striatal neurons entering the SVZ and making functional connections leading to increased calcium activity in SVZ cells (illustrated in diagram of Figure 9).

We identified processes from striatal neurons projecting along and into the SVZ. The majority of neurons contacting SVZ cells were medial spiny neurons (80%) and a minority (20%) were aspiny neurons. We found both dendrites and axons entering the SVZ. The presence of axons fits with the identification of neurofilament and VGAT staining in the SVZ. There are some precedents for neuronal processes in the SVZ. Axons had been observed in the SVZ using electron microcopy (Peretto et al., 1999; Mercier et al., 2002). Dopaminergic fibers from the substantia nigra have also been found to project axonal terminals into the SVZ. Nitrergic neurons (NADPH-diaphorase positive) in the striatum were found to border the SVZ and occasionally send nitrergic axons intermingled with chains of neuroblasts (Moreno-Lopez et al., 2000). The identification of dendrites in our study was unexpected. We could not determine whether one process type was more predominant than the other for technical reasons. The visualization of axons was limited due to their small size and the fact that axons were often physically cut by going in and out of the slices. The application of novel technology allowing clearing of the brain could be used in thicker sections to better address this issue in future experiments. We found that striatal neurons send 3–4 processes into the SVZ that covered a mean of 56 μm in the SVZ. Although this seems limited, the SVZ is a small region (about 50 μm wide on the lateral side). In addition, since SVZ cells are in close proximity to each other, the release of diffusible factors from a single, activated cell could readily activate surrounding cells. This is also true for calcium activity that could travel between cells as discussed below.

Using transgenic mice, we found that dendrites project toward neuroblasts, but do not appear to directly contact them. Processes projected to or crossed paths with processes or soma of GFAP-expressing cells, some of which are neural progenitor cells. Although neuronal and GFAP^+^ processes appeared to overlap, ultrastructural analysis would be required to further examine the distance between these processes and identify the type of contact. In addition, we did not explore connection to other cell types such as transit amplifying cells.

These findings were obtained between P14 and P25. We have not explored in younger animals. The cytoarchitecture of the SVZ is dramatically different and the SVZ is much wider and progressively changes to its near mature structure by the end of the second postnatal week (Peretto et al., 2005). We thus expect our findings to be applied in fully adult mice. In neonate animals, GFAP^+^ cells of the SVZ are radial glia and send long processes throughout the striatum. It is thus highly conceivable that striatal neurons contact the radial glia processes even without entering the SVZ, but this needs to be further explored.

In an effort to determine whether these neuronal processes had any functional outcome on SVZ cells, we performed calcium imaging. We found that depolarizations from a single neuron were sufficient to trigger calcium activity in SVZ cells. Using bulk stimulation, which was more reproducible, we identified that neuron depolarizations led to GABA release and GABA_A_R-mediated calcium increase consistent with the finding that striatal neurons are essentially GABAergic. In calcium recording experiments, some cells responded quickly to the neuronal stimulation (e.g., Figure 7G) while others responded with some lag (e.g., Figure 7F). To understand this finding, it is important to know that not all SVZ cells are loaded with the calcium dyes (Lacar et al., 2010) and that calcium increase in GFAP^+^ cells leads to calcium waves that propagate to 10–15 other cells (Lacar et al., 2011). Thus, for analysis of calcium activity, we may miss some cells that may be directly contacted by neuronal processes; in addition, cells not contacted may respond as part of a calcium wave or a secondary response due to either GABA or glutamate/ATP released from neuroblasts and GFAP^+^ cells, respectively (Liu et al., 2005; Platel et al., 2010; Lacar et al., 2012b).

Regarding baseline calcium activity, we previously reported that the GABA_A_R blocker, bicuculline, significantly decreased the frequency of calcium transients in SVZ cells, in particular GFAP^+^ cells (Young et al., 2010). However, we found that TTX had no effect on baseline calcium activity possibly due to the well-documented low firing rate of medium spiny neurons in slices as well as *in vivo* in resting state.

How do these new findings integrate into the already reported GABAergic signaling in the SVZ? In the SVZ, GABA is released from two sources, neuroblasts (Liu et al., 2005) and striatal neurons (shown here), and depolarizes both neuroblasts and GFAP^+^ cells via GABA_A_R activation (Wang et al., 2003; Liu et al., 2005; Young et al., 2012) due to the presence of Cl^−^ influx Na-K-2Cl co-transporter (NKCC1) and the absence of a Cl^−^ efflux K-Cl co-transporter (KCC2) in both neuroblasts (Mejia-Gervacio et al., 2011) and GFAP^+^ cells (unpublished observations, Henschel and Bordey). GABA_A_ action on SVZ cells remains depolarizing independent of the age of the animals, but becomes hyperpolarizing in neuroblasts when they enter the olfactory bulb network (Young et al., 2012). In other regions including the striatum, GABA_A_ action changes from depolarizing to hyperpolarizing during the first two postnatal weeks (Ben-Ari and Spitzer, 2010; Ben-Ari et al., 2012).

GABA release from neuroblasts is non-vesicular and produces a tonic activation of GABA_A_Rs in neuroblasts (Bolteus and Bordey, 2004) and GFAP^+^ cells (Liu et al., 2005) leading to a tonic depolarization. A tonic depolarization can lead to either an increase in the frequency of calcium transients as shown in neuroblasts (Gascon et al., 2006) or a tonic influx of calcium in particular in GFAP^+^ cells because they express low threshold voltage-activated T-type calcium channels (Young et al., 2010). Regarding the neuronal source of GABA, we do not know whether dendrites or axons or both contribute to GABA release, but we propose that an axonal release is occurring since VGAT immunostaining was observed in the SVZ and dendrites of striatal neurons are not known to have vesicular GABA release. An action-potential dependent vesicular GABA release from neurons is expected to generate larger depolarizations than tonic activation and thus lead to calcium transients as observed in Figure 7G. Considering that our calcium loading protocol preferentially labels GFAP^+^ cells vs. neuroblasts (Lacar et al., 2011, 2012b), we anticipate that cells responding to neuronal stimulation are GFAP^+^ cells and that the calcium transients involve L type calcium channels (Young et al., 2010). Thus, neuroblast- and neuronal-released GABA leads to tonic and phasic depolarizations, respectively, and are expected to activate different types of calcium channels in GFAP^+^ cells that may differentially affect their behavior.

Inhibition of GABA_A_R activity with bicuculline in acute slices led to increased GFAP^+^ cell/NPC proliferation (Liu et al., 2005). This effect is likely due to inhibition of tonic GABA_A_R activation because TTX (action potential)-dependent phasic activation had no effect on calcium activity in SVZ cells. By contrast, knockdown of NKCC1, which occluded applied GABA-induced calcium increase in NPCs, decreased NPC proliferation *in vivo* (Young et al., 2012), suggesting that a phasic activation of GABA_A_Rs promotes NPC proliferation. Considering the spread of neuronal inputs into the SVZ, the phasic GABA release from neurons could synchronize NPC proliferation and calcium activity, which has been shown to regulate blood flow regulation. The striatal input to the SVZ may thus act as a coordinator of SVZ cell proliferation and nutrient uptake from the blood, although this remains to be fully examined.

Our findings provide a mechanism for brain activity-coupled neurogenesis in the SVZ. Indeed, brain activity has been shown to regulate SVZ cell proliferation (Parent et al., 2002, 2006; Geraerts et al., 2007; Aponso et al., 2008) [for review see (Young et al., 2011)] and the striatum receives massive inputs from the cortex, ventral tegmentum, globus pallidus, and substantia nigra. It is nevertheless important to mention that the proposed GABAergic signaling from striatal neurons to SVZ cells may have a greater functional significance in disease state. Indeed, in resting state, medium spiny neurons have a low firing rate. But in disease condition such as in Parkinson's disease, it has been shown that striatal neurons firing rate significantly increases and synaptic function is altered (Hammond et al., 2007; Picconi et al., 2012) and may thus affect SVZ cell behavior such as proliferation. The ultimate effect on proliferation may, however, be a combination of several mechanisms considering that in Parkinson's disease SVZ cell proliferation is significantly decreased and dopamine itself affects SVZ cell proliferation (Baker et al., 2004; Hoglinger et al., 2004).

Collectively, our study identifies a novel signaling modality involving GABA_A_R activation on SVZ cells and emphasizes the complexity of the signaling network in this region that regulates the behavior of SVZ cells.

## Conflict of interest statement

The authors declare that the research was conducted in the absence of any commercial or financial relationships that could be construed as a potential conflict of interest.

## Supplementary material

Figure S1 **(A)** Images of IR-DIC without and with CellTracker orange loading the SVZ and proximal striatal neuron. A patch pipette is attached to a striatal neuron. **(B)** Images of single optical sections at different depths going from top to bottom of the slice. A striatal neuron was filled with Alexa Fluor-488. **(C)** 3D-surface reconstructions and one IR-DIC optical section with Imaris in the X-Y and X-Z plans. **(D)** Maximum intensity projection in Imaris with red overlay to illustrate the extent of processes in the SVZ and bordering the SVZ in blue.
