# Supplementary figures and images for: GABAergic striatal neurons project dendrites and axons into the postnatal subventricular zone leading to calcium activity
Source: Front Cell Neurosci. 2014 Jan 28;8:10. doi: 10.3389/fncel.2014.00010 (PMC3904109; doi:10.3389/fncel.2014.00010)

Figure S1, Young

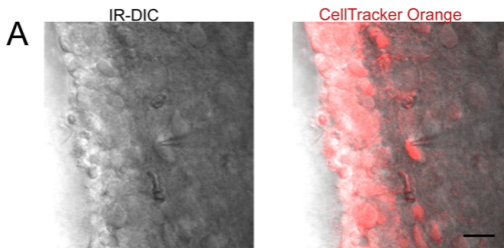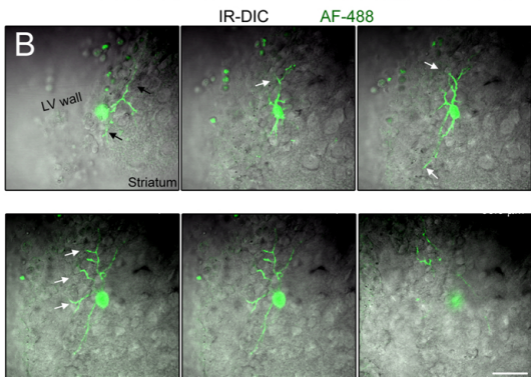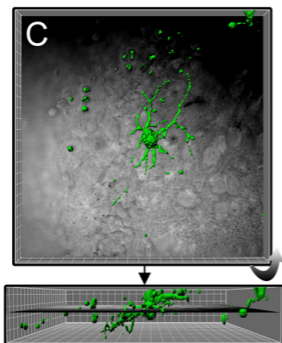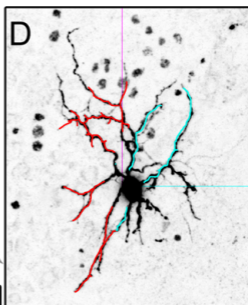

Supplement: Figure S1 — (A) Images of IR-DIC without and with CellTracker orange loading the SVZ and proximal striatal neuron. A patch pipette is attached to a striatal neuron. (B) Images of single optical sections at different depths going from top to bottom of the slice. A striatal neuron was filled with Alexa Fluor-488. (C) 3D-surface reconstructions and one IR-DIC optical section with Imaris in the X-Y and X-Z plans. (D) Maximum intensity projection in Imaris with red overlay to illustrate the extent of processes in the SVZ and bordering the SVZ in blue. [file Presentation1.PDF]
